# Adhesion, Vitality and Osteogenic Differentiation Capacity of Adipose Derived Stem Cells Seeded on Nitinol Nanoparticle Coatings

**DOI:** 10.1371/journal.pone.0053309

**Published:** 2013-01-07

**Authors:** Sarah Strauß, Anne Neumeister, Stephan Barcikowski, Dietmar Kracht, Jörn W. Kuhbier, Christine Radtke, Kerstin Reimers, Peter M. Vogt

**Affiliations:** 1 Department for Plastic- Hand- and Reconstructive Surgery, Hannover Medical School, Hannover, Germany; 2 Laser Zentrum Hannover e.V., Hannover, Germany; 3 Technical Chemistry I and Centre for Nanointegration, University of Duisburg-Essen, Essen, Germany; University of California San Diego, United States of America

## Abstract

Autologous cells can be used for a bioactivation of osteoimplants to enhance osseointegration. In this regard, adipose derived stem cells (ASCs) offer interesting perspectives in implantology because they are fast and easy to isolate. However, not all materials licensed for bone implants are equally suited for cell adhesion. Surface modifications are under investigation to promote cytocompatibility and cell growth. The presented study focused on influences of a Nitinol-nanoparticle coating on ASCs. Possible toxic effects as well as influences on the osteogenic differentiation potential of ASCs were evaluated by viability assays, scanning electron microscopy, immunofluorescence and alizarin red staining. It was previously shown that Nitinol-nanoparticles exert no cell toxic effects to ASCs either in soluble form or as surface coating. Here we could demonstrate that a Nitinol-nanoparticle surface coating enhances cell adherence and growth on Nitinol-surfaces. No negative influence on the osteogenic differentiation was observed. Nitinol-nanoparticle coatings offer new possibilities in implantology research regarding bioactivation by autologous ASCs, respectively enhancement of surface attraction to cells.

## Introduction

Bone tissue engineering is an attractive field with regard to clinical application to bridge bone tissue defects e.g. after tumor resection. Engineering of a piece of bone, as it is needed for bone substitution in bigger defects, is impossible in present state of scientific and technical knowledge. Furthermore, time needed for bone engineering in vitro makes a usage for acute incidences complicated. Up to now, large bone defects are treated by dense implants or autologous transplants, e.g. solid grafts from the fibula or cancelous bone from the iliac crest, with resulting donor morbidity. Nevertheless, withdrawal of autologous transplants is a serious load for the patient while synthetic implants made from dense metal or ceramics often are accompanied with a lack of osseointegration [1].

In this context Nitinol (NiTi) – an alloy consisting of nickel and titanium – is a promising material for dense implants. Concerning flexibility and stability NiTi shape memory alloys (SMAs) show bone related characteristics, which render them predestinated as an implant material [2], [3], [4], [5].

However, fast and stable osseointegration still remains a challenge. Particularly, integration of plane surfaces is often inadequate. Many studies are focusing on the surface characteristics and their optimization. It has been shown that rough surfaces are advantageous for cell adhesion. Especially nanostructuring of surfaces can enhance cell adhesion and thereby improve osseointegration of the implant adhesion 1,6,7,8,9.

In this context, surface coating using nanoparticles is a simple method to create a nanoscaled surface structure [10]. In order to prevent unwanted physical and/or chemical interactions between surface and nanoparticles, usage of similar materials for both is a promising option. A study with human primary endothelial and smooth muscle cells regarding the cytotoxicity of NiTi-nanoparticles revealed that potential toxicity depends partly on the additive used to stabilize nanoparticle colloids [11]. In combination with an appropriate additive colloid nanoparticles (unbound nanoparticles) have to be concentrated 6 times higher than needed to coat an area with a nanoparticle monolayer covered by a single cell to reach toxic levels. So even in the unlikely case of a complete detachment of nanoparticles from the coating of the implant the nanoparticle concentration can not reach a level which is toxic to the cells [12].

Another strategy to enhance implant integration exploits the bioactivation of surfaces by preseeding with autologous mesenchymal stem cells (MSCs) from the bone marrow. In vivo these are the source of cells differentiating to osteoblasts which build the bone. Studies of Bruder et al. showed an enhanced osseointegration of ceramic implants which had been preseeded with autologous MSCs [13]. It was recently demonstrated that MSCs from bone marrow aspirates adhere and differentiate osteogenically on NiTi [14], [15].

While for MSC isolation a bone marrow aspirate or a biopsy has to be taken, with all its risks for the patient (e.g. osteomyelitis), adipose derived stem cells (ASCs) can be isolated easily in considerable numbers from subcutaneous fat. 1 g of adipose tissue yields approximately 5×10^3^ stem cells, which is 500 times greater than the number of MSCs in 1 g of bone marrow [16].

ASCs are able to differentiate osteogenically [18], [19] and therefore are a promising alternative to MSCs regarding the preseeding of implant surfaces. ASCs were already applied successful for the clinical treatment of wounds or contour defects [20]. In a case report, Lendeckel et al. successfully treated a calvarial bone defect with ASCs [21].

In summary, the ideal NiTi-osteoimplant can be bioactivated with ASCs. As roughness is an important factor for cell adhesion and osseointegration [1], [6], [7], [8], [9] a coating utilizing NiTi-nanoparticles could ameliorate the attachment of cells on the implant.

In previous studies Barcikowski et al. showed that ASCs are able to grow on cover glasses coated with NiTi-nanoparticles [22]. Although their data indicated that NiTi-nanoparticles do not exert a negative influence on ASCs, cytocompatibility has not been studied in detail yet. For that reason the interactions of these cells with NiTi-nanoparticles still remain unclear. The study at hand analyzes adhesion, cell viability, growth and proliferation as well as osteogenic differentiability of ASCs on coatings with NiTi-nanoparticles. Here, human ASCs were seeded on NiTi-sheets which were half-side coated with NiTi-nanoparticles by two different coating methods (see methods part) and additionally, on NiTi-coated and uncoated cover glasses. In order to exclude possible adverse nanoparticle-related effects, cell reactions on colloidal NiTi-nanoparticles were investigated as well.

Cell viability was evaluated by life/dead assay. Metabolic activity and adhesion were determined. Moreover, cell morphology and osteogenic differentiation were analyzed by scanning electron microscopy and immuno- and histochemical stainings.

## Methods

### ASC-isolation and cultivation

Human ASCs were isolated from fat tissue obtained from eleven male and female donors aged between 16 and 40 years after obtaining the written informed consent from the patients following ethic standards as described previously [16], [17]. The obtained tissue was used anonymously. Fat was separated from the dermo-epidermal layer, minced with sterile scissors followed by enzymatic digestion with 0.075% collagenase CLS (Biochrom AG, Berlin, Germany) in PBS (Biochrom) for 30 minutes at 37°C under permanent shaking. Cell containing liquid was centrifuged at 1.200× g at room temperature for 10 minutes. Cells were cultivated in DMEM/Hams-F12 (PAA) with 10% fetal calf serum (FCS) (Biochrom), 50 U/ml penicillin/streptomycin (Biochrom), 10 ng/ml FGF and 50 µg/ml ascorbate-2-phosphate (Sigma Aldrich, Taufkirchen, Germany) at 37°C and 5% CO_2_. For analyses cells in passage 3 to 5 were seeded on cover glasses and Nitinol-surfaces with and without nanoparticle-coatings. Cultivation time varied according to analyses between 24 hrs, 48 hrs and 6 weeks (for osteogenic differentiation).

### Osteogenic differentiation

Osteogenic differentiation was induced by application of dexamethasone and glycerol-2-phosphate disodium salt. Accordingly cells were cultivated up to 6 weeks in DMEM/Hams-F12 with 10% FCS, 50 U/ml penicillin/streptomycin, 50 µg/ml ascorbate-2-phosphate, 39.3 ng/ml dexamethasone (Sigma Aldrich, Taufkirchen, Germany) and 0.756 mg/ml glycerol-2-phosphate disodium salt (Sigma Aldrich, Taufkirchen, Germany). Medium was changed three times per week.

### RAW264.7 cultivation

The murine macrophage cell line RAW264.7 (ATCC, Manassas, USA) was cultivated with DMEM high glucose (PAA) containing 10% FCS, 50 U/ml penicillin/streptomycin and 0.1 mg/ml sodium-pyruvate (PAA) at 37°C and 5% CO_2_.

### Nanoparticle production

NiTi- (average hydrodynamic diameter of 60 nm), Ti- and Ni- and Ag-nanoparticles were produced using laser ablation in liquids as previously described [22]. The pulse energy of the femtosecond laser was 200 µJ and the laser beam speed rate 1 mm s^−1^. Laser ablation was carried out in double distilled aqueous solution of citrate or cysteine, both with a final concentration of 5 mM. Nanoparticle concentration ranged from 80 to 282 µg/ml due to the fabrication processes. Dispersions were diluted with distilled water to 2 µg/ml for cell viability tests.

### Surface coating

#### Depending on the requirements of analyses two different coating methods were used

While homogenous coatings could be deposited on conductive surfaces by electrophoretic deposition, drying allowed deposition of defined quantities of nanoparticles on non-conductive surfaces like cover glasses.

Electrophoretic deposition of nanoparticles was done by applying voltage (5 V per cm for 1 h) to two NiTi-sheets (Alloy M, Memory-Metalle GmbH, Weil am Rhein, Germany) which were aligned in parallel order and immersed in the nanoparticle dispersion with a distance of 1 cm between the sheets. By application of voltage the two sheets worked as electrodes. As the colloidal NiTi-nanoparticles have a negative zeta-potential they move through the electric field to the anode and deposit there [12].

Deposition by drying was carried out by dropping defined concentrations (10 to 100 µg/cm^2^) of colloidal nanoparticles on “activated” (pretreated with nitric acid) cover slips. Subsequently, samples were air-dried. While the solvents disappeared, nanoparticles were left as a coating on the surface [12].

All NiTi-sheets used were martensitic and did not change to austenitic phase when sterilized by autoclaving (according to manufacturer's data). Sheets were produced by rolling, so surfaces showed the typical structure with small furrows.

### Cell viability

Cell viability was tested by live/dead assay (Invitrogen, La Jolla, USA, #L3224) and cell titer blue assay (Promega, Mannheim, Germany, #8082).

For live/dead assay ASCs were seeded on cover glasses and NiTi-surfaces with or without NiTi-, Ni-, Ag- and Ti-nanoparticle coatings. To analyze the influence of the materials on undifferentiated ASCs, cells were cultivated for 24–48 hrs on the respective surfaces. Osteogenically differentiated cells were analyzed after 6 weeks of cultivation with osteogenic medium. To perform the assay, cells were incubated with commercially available live/dead solution for 30 min and then analyzed by fluorescence microscopy (Olympus CK40, Cell D). Living cells were stained green by calcein, dead cells red by ethidium-homodimer-1.

Cell titer blue assays were performed in 96-well plates. 10^5^ cells per well were seeded in duplicates on coated and uncoated NiTi surfaces and incubated for 48 hrs. To analyze effects of colloidal nanoparticles 10000 cells per well were seeded and incubated for 24 hrs. Colloidal NiTi, Ti-, Ni-nanoparticles in H_2_O, 5 mM citrate or 5 mM cysteine were added to 2 µg/ml as final concentrations of nanoparticles. Solutions of 5 mM citrate or 5 mM cysteine without nanoparticles served as controls. After 24 and 48 hrs incubation cell titer blue solution was added and incubated at 37°C for 4 hrs. Cells metabolize resazurine via reduction to resorufin which can be excited to fluorescence at 590 nm. The produced fluorescence intensity is thus proportional to the number of viable cells. Fluorescence was measured with a Tecan GENios multiwell reader (Tecan, Männedorf, Swiss).

### Statistical analyzes

Statistical analyzes were performed with Graph Pad Prism Software. To check statistical significance of the measured metabolic activities (see above) one way ANOVA, followed by Dunnetts post hoc test were carried out.

### Immunofluorescence

Immunofluorescence was performed on NiTi-nanoparticle coated and uncoated cover glasses. 10^5^ cells were seeded on cover glasses and incubated for 48 hrs and up to 6 weeks and then fixed with 4% (w/v) paraformaldehyde (Sigma Aldrich, Taufkirchen, Germany) for 20 min, blocked and permeabilized for 3×5 min with PBS containing 0.1% TritonX-100 and 1% BSA. Primary antibodies against integrins α_5_ (Chemicon #MAB1978) and ß_1_ (Chemicon #MAB1959), fibronectin (Acris #R1154PX, according to manufacturers data no cross reactions to other species), tubulin (abcam #ab6160-100), bone alkaline phosphatase (abcam, #ab108337, ab17272), osteocalcin (abcam, #ab13420), tubulin (abcam #ab6160) and the actin cytoskeleton marker phalloidine-FITC (Invitrogen #F432) were used in dilutions of 1∶500 and incubated at room temperature for 1 h. The samples were washed with PBS containing 0.1% TritionX-100 and 0.1% BSA. Secondary antibodies coupled to Alexa 546 (Invitrogen #A11056, A10036, A11060) and Alexa 488 (Invitrogen #A21200, A11059, A21441) were used at dilutions between 1∶1000 and 1∶4000. Samples were washed several times with PBS w/o Ca. Nucleic staining was performed with DAPI included in the mounting medium (Vectorshield #H-1200). Samples were analyzed by fluorescence microscopy (Zeiss Axiovert 200M, AxioVision; Olympus SZX16, Cell F).

### Alizarin red staining

Calcium deposits of cells, which are characteristics of bone tissue, were detected by alizarin red staining. ASCs were seeded on NiTi-sheets and coated cover glasses. Osteogenic differentiation was induced by culture media for 6 weeks. Samples were fixed with 3.7% formalin. Specimens were rinsed with tab water for 1 h and washed with 0.1 M boric acid (pH 4). Afterwards they were incubated in alizarin red solution (0.5%, pH 4, A-5533, Sigma Aldrich) for 1 h, rinsed with boric acid, washed with distilled water, rinsed with 95% ethanol and then air dried. Samples were analyzed by reflected-light microscopy (Olympus SZX16, Cell F) and digital microscopy (VK-9700 and VHX, Keyence with VK-Analyzer).

### Scanning electron microscopy

Cells on scaffolds were cultured for 24 hrs and up to 6 weeks (osteogenic differentiation). Samples were fixed in 0.2 M Na-cacodylate buffer (pH 7.4) with 2.5% glutaraldehyde for 24 hrs, followed by dehydration with acetone (30%, 50%, 70%, 9 0%, 100%) for 3×10 min. Critical point drying was performed with acetone/CO_2_ in CPD 030 system (Bal-Tec, Balzers, Liechtenstein). Samples were sputtered with gold (Sem Coating System, Polarion). Scanning electron microscopy was done with a SEM 505 (Philips). Pictures were taken with SEM-Software by Preiss and Gebert [23].

## Results

### NiTi-nanoparticles are not toxic to ASCs and RAW264.7 cells

In order to rule out any harmful influences of NiTi-nanoparticles on ASC growth and vitality a number of assays were performed.

**Figure 1:**
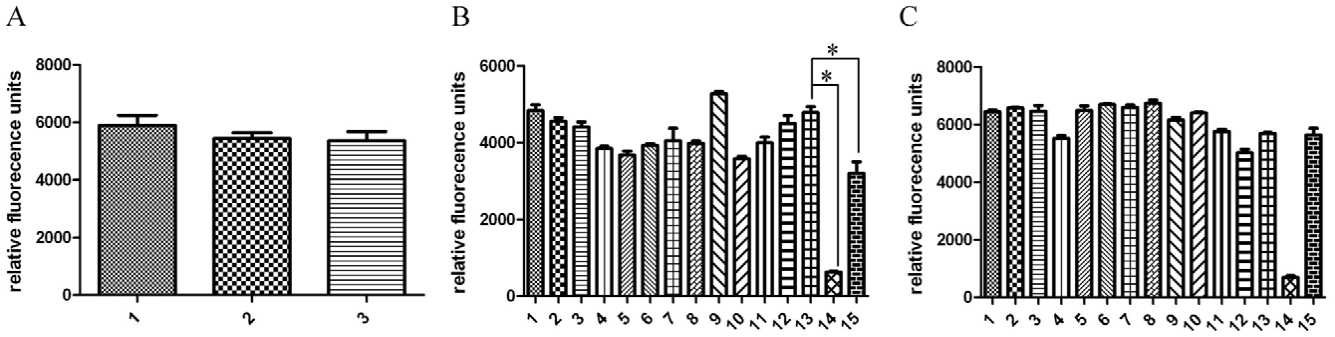
Metabolic activity after 48 hrs stimulation. A: ASCs on NiTi sheets (1), NiTi-nanoparticle coated NiTi-sheets (2) and cell culture plastic (3) showed no significant differences in metabolic activity. B: ASCs incubated with nanoparticle colloids and/or additives on cell culture plastic (1–14) and Nickel sheets (15). C: macrophages incubated with nanoparticle colloids and/or additives on cell culture plastic (1–14) and Nickel sheets (15). B & C: 1: H2O, 2 Citrate (Ci), 3 Cystein (Cy), 4 NiTi H2O, 5 NiTi Ci, 6 NiTi Cy, 7 Ti H2O, 8 Ti Ci, 9 Ti Cy, 10 Ni H2O, 11 Ni Cy, 12 Ni Cy, 13 cell culture medium, 14 DMSO, 15 Ni sheet. Statistical analysis of metabolic assay was performed with graph pad prism software by using one way ANOVA, followed by Dunnetts post hoc test. Only control without stimulation (B13) compared to DMSO control (B14) and stimulation with nickel (B15) showed significant changes in metabolic activity (indicated by asterisk).

We first compared NiTi nanoparticle bound to NiTi-sheets and uncoated sheets to cells kept on cell culture plastic surfaces. ASCs showed no significant difference on NiTi-nanoparticle-coated and uncoated NiTi-surfaces after 48 hrs of stimulation, when their metabolic activity was measured with CellTiterBlue assay (Fig. 1A).

The influence of unbound nanoparticles was investigated by measurements using colloids. Additionally, cysteine and citrate solutions (5 mM) without nanoparticles were also included as controls since these additives were used as stabilization agents during nanoparticle synthesis. Neither colloidal nanoparticles at their highest concentration of 2 µg/ml nor their controls did exert a significant negative influence on ASCs' metabolic activity within 48 hrs. 10% DMSO and Ni-sheets, which are toxic to cells, served as positive controls and caused a significant loss of metabolic activity as the cells died (Fig. 1B).

A loss of activity of cells which were cultivated with Nitinol would have implicated a toxic effect of the alloy. As results were comparable to cells grown under normal cell culture conditions one can conclude that Nitinol in the tested forms has no toxic influence on metabolic activity of ASCs (Fig. 1A and B).

Murine macrophages were checked for metabolic activities after 48 hrs to get hints on possible immune reactions to the presence of NiTi-nanoparticles. No significant enhancement or decrease of cell metabolism was observed for all materials and solvents tested. A reaction to the tested concentration of colloidal nanoparticles would have implied an activation of these macrophages or cell death with loss of metabolic activity. As activation or loss of activity did not occur, we assessed the tested nanoparticle-concentration as uncritical for macrophages in vitro. (Fig. 1C)

### Cell adhesion on NiTi-nanoparticle-coated surfaces as a prerequisite of osteogenic differentiation

In a first series of experiments NiTi nanoparticle coated cover glasses were seeded with ASCs for 48 hrs. Cover glasses were treated as described above to achieve a coating density of 50 µg/cm^2^ and 100 µg/cm^2^.

**Figure 2:**
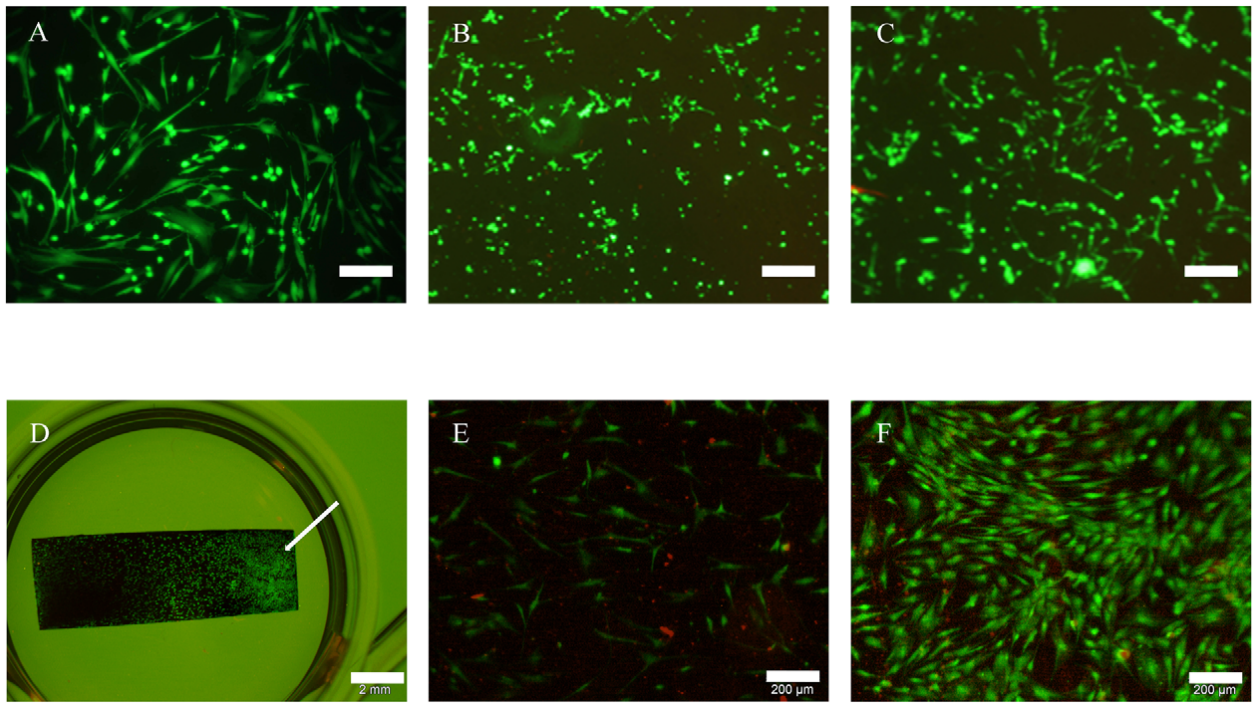
Live/dead assay after cultivation of ASCs for 48 hrs; green: vital cells; red: dead cells. Cells showed typical morphology in all approaches of which representative examples were shown. Abnormal rates of dead cells were not observed. A: cover glass, scale bar 200 µm. B: cover glass coated with 50 µg/cm^2^ NiTi-nanoparticles, scale bar 200 µm. C: cover glass with coated with 100 µg/cm^2^ NiTi-nanoparticles, scale bar 200 µm. D: NiTi-sheet with partial coating of 300 µg/cm^2^ NiTi-nanoparticles (indicated by arrow), scale bar 2 mm. E: detail of NiTi-sheet without coating, scale bar 200 µm. F: detail of NiTi-sheet coated with 300 µg/cm^2^ NiTi-nanoparticles, scale bar 200 µm.

Cells had the typical spindle-shaped morphology on uncoated glass controls and were vital to a large extent (Fig. 2A). On coated specimen (Fig. 2B and C) cell attachment appeared punctual in dependence of coating density in microscopic visualization. Green fluorescence, however, indicated that cell viability was unaffected by glass treatment. Interestingly, the non-fluorescent spaces between the cells were reduced in correlation with increasing nanoparticle concentration so cells appeared more confluent on coated surfaces in the first 48 hrs (Fig. 2). Even on Ni- and Ag-nanoparticle coated cover glasses the relative amounts of vital ASCs have been found to be higher than 50% (data not shown) although both types of nanoparticles are known for toxic effects in different settings [11], [12].

Furthermore, we analyzed the effect of NiTi nanoparticle coating on cell attachment. To this end, we coated NiTi-sheets with NiTi nanoparticles via electrophoretic deposition. The sheets were partially coated, while one half was left without particles the other was coated with 30 to 300 µg/cm^2^. Figures 2D, 2E, 2F show representative results obtained with a concentration of 300 µg/cm^2^. A cell suspension of ASCs was added to the sheets and covered with access volume of medium. Cellular attachment preferentially occurred on the coated half of the sheets (Fig. 2D). Cells grew to a much lesser extent on uncoated parts and several dead cells were observed as indicated by red nuclei (Fig. 2E). The common spindle-like morphology was observed by reflected light microscopy on uncoated and coated parts (Fig. 2F). This indicates that the punctual appearance of the samples of cover glasses was caused by loss-of-signal due to nanoparticles.

Nevertheless, we further characterized ASCs adhesion on NiTi-nanoparticle coated cover glasses by several immunostainings. This time a higher resolution epifluorescence was chosen to allow the full observation of cytoskeleton organization. Signal-loss due to nanoparticles was overcome by prolonged exposure times.

**Figure 3:**
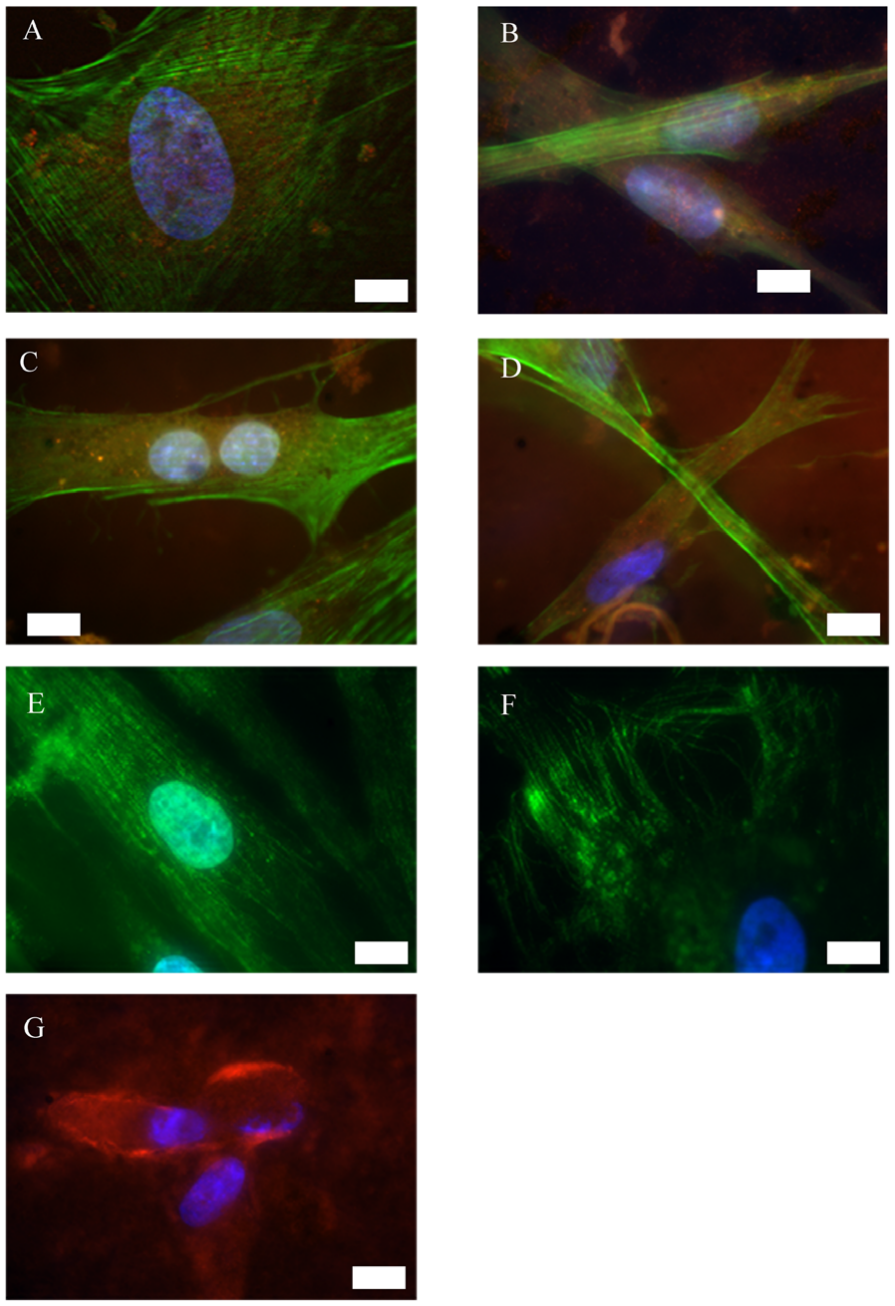
Immunofluorescence of undifferentiated ASCs on cover glasses with or without NiTi-nanoparticle coating of 50 µg/cm^2^. Actin and tubulin arrangement is comparable on uncoated and coated glass. Integrins α_5_ and ß_1_ are involved in adhesion on both surface types. Cells secrete fibronectin as part of the extracellular matrix. A: uncoated glass; blue: nuclei, green: actin, red: integrin α_5_, scale bar 10 µm. B: coated glass; green: actin, red: integrin α_5_, scale bar 20 µm. C: uncoated glass; blue: nuclei, green: actin, red: integrin ß_1_, scale bar 20 µm. D: coated glass; blue: nuclei, green: actin, red: integrin ß_1_, scale bar 20 µm. E: uncoated glass; blue: nuclei, green: tubulin, scale bar 20 µm. F: coated glass; blue: nuclei, green: tubulin, scale bar 20 µm. G: coated glass; blue: nuclei, red: fibronectin, scale bar 20 µm.

Integrins α_5_ and ß_1_ play a role for osteogenic differentiation of MSCs [6]. We assume that ASCs may behave similar to MSCs, which were already shown to differentiate osteogenic on untreated NiTi [14], [15]. These integrins were found to be part of the focal adhesion points of ASCs in the NiTi-nanoparticle group (Fig. 3B, 3C, 3D) as well as in the control group on uncoated cover glasses (Fig. 3A and C) as indicated by spots of red fluorescence. Arrangement of actin cytoskeleton (green fluorescence) was found to be homogenous and did also not differ from controls; no stress fibers were visible in any specimen. Tubulin fibers also appeared regularly (Fig. 3E and F). All immunofluorescence labelings did not indicate visual differences in cell organization or growth behavior in dependence on the surfaces compared, but the cells adhered efficiently on all NiTi nanoparticle coated surfaces.

Additionally, extracellular matrix secretion was determined. ASCs typically secrete fibronectin as the most prominent part of their extracellular matrix when fully attached to a rigid surface. High amounts of fibronectin were secreted by ASCs grown on NiTi-nanoparticle coated cover glasses (Fig. 3G) and similarly on coated NiTi-surfaces (data not shown).

For all immunofluorescence stainings as an additional negative control nanoparticle-coated surfaces (glass and NiTi) without cells were treated after the same protocol to rule out false results due to unspecific adherence of antibodies to the coating. No unspecific binding of secondary antibodies occurred under the chosen conditions.

All in all no evidence was found that NiTi-nanoparticles have a negative influence on ASCs' adhesion or the organization of their cytoskeleton as these are important factors for cell motility and osteogenic differentiation capacity.

### ASCs' confluence and dimensional growth appear to be higher on NiTi-nanoparticle coated surfaces

**Figure 4:**
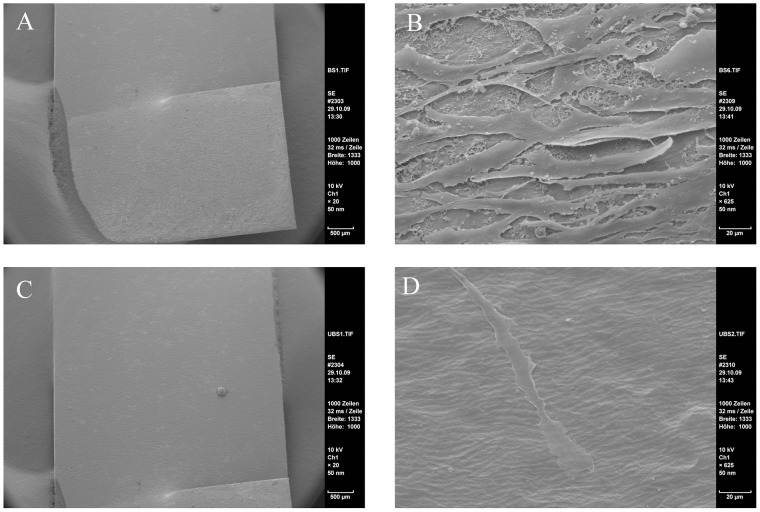
SEM pictures of ASCs on NiTi for 48 hrs. More cells were found on the coated parts of the sheets. There grew with a dimensional shape whereas only few cells were found on the uncoated parts. There cells appeared in a very flat shape. A: NiTi-nanoparticle coated part, overview; scale bar 500 µm. B: coated part, detail; scale bar 20 µm. C: uncoated part, overview; scale bar 500 µm. D: uncoated part, detail; scale bar 20 µm.

As cell distribution appeared more confluent on coated surfaces in live/dead stainings, we analyzed their growth pattern in a higher resolution in the next step. To this end, samples of partly coated NiTi-sheets seeded with ASCs were visualized by SEM. As a result, we were able to confirm our previous findings by SEM analyzes as such the cells attached densely on the coated part of the sheet (Fig. 4A and 4B) while found sparsely on the bare material (Fig. 4C and 4D). Furthermore, we could observe that ASCs grew not only in dense layers but showed 3-dimensional structures while cells on uncoated parts appeared flat shaped (Fig. 4B and 4D).

### Osteogenic differentiation of ASCs on NiTi-nanoparticle coating

**Figure 5:**
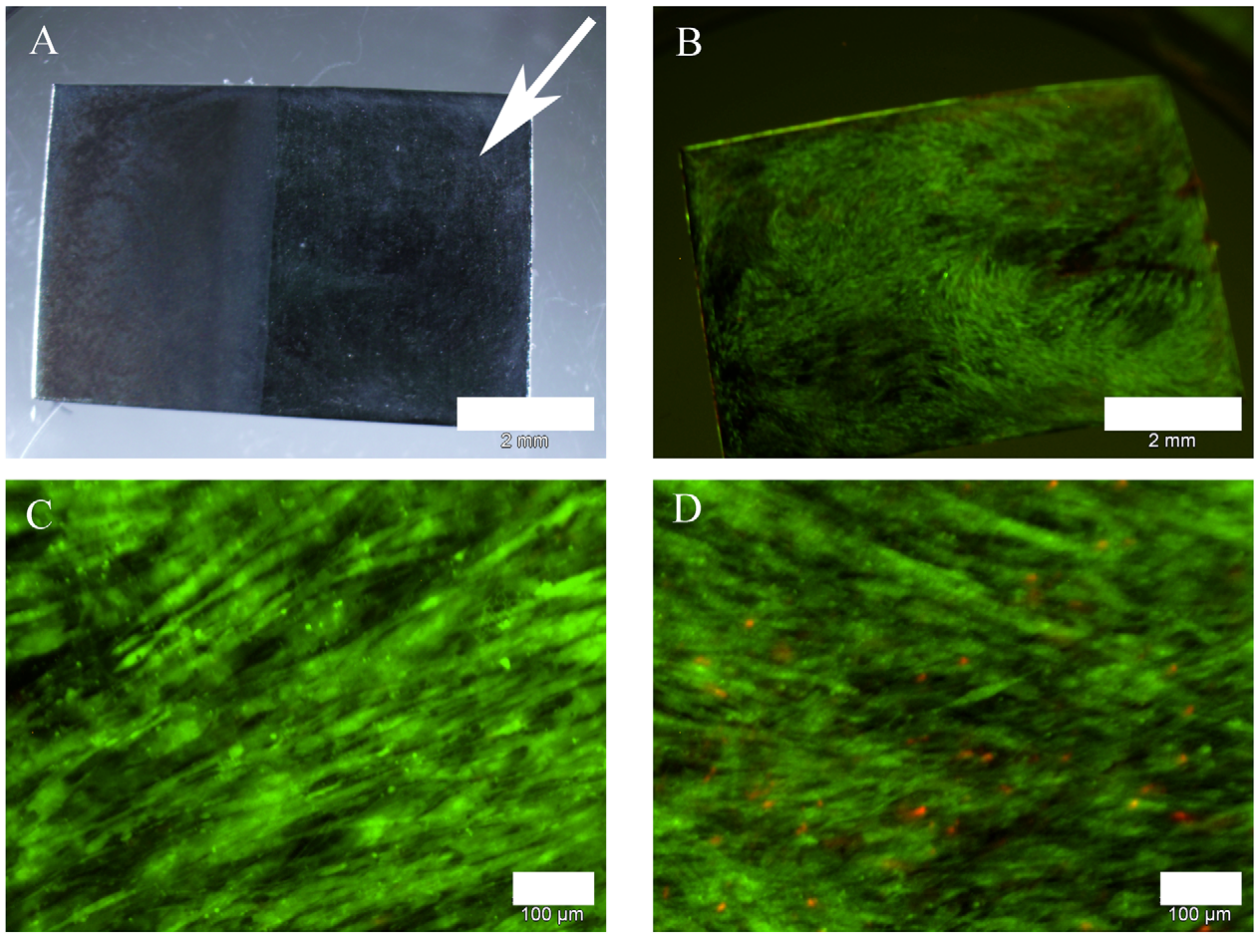
Osteogenic ASCs on NiTi-sheet, half-coated with NiTi-nanoparticles. Light microscopic overview shows no visual differences between coated and uncoated parts, whereas live/dead stainings revealed higher rates of vital cells on coated parts. A: light microscopic overview, coated part indicated by arrow; scale bar 2 mm. B: specimen of part A in fluorescence; green: vital cells, red: dead cells; scale bar 2 mm. C: coated part, detail; green: vital cells, red: dead cells; scale bar 100 µm. D: uncoated part, detail; green: vital cells, red: dead cells; scale bar 100 µm.

Taking into concern our primary intention to evaluate suitability of NiTi nanoparticles for optimization of NiTi bone implants, the impact of particle coating on the osteogenic differentiation capacity of ASCs was analyzed. Therefore, samples were treated with osteogenic differentiation medium for up to six weeks. We first analyzed the effect of long-term exposure to NiTi and NiTi nanoparticles by life/dead assay. Although the differences in cell density between the two parts of the sheets (Fig. 5A) were still visible they were less striking than at the short time culture. Nevertheless, cells grew still with a higher density on the coated part and in growth pattern typical for osteogenic differentiation (Fig. 5B). Detailed microscopy showed that there were also differences in cellular vitality. While there could be observed practically no reddish nuclei in the coated part of the samples (Fig. 5C), the uncoated part was characterized by appearance of dead cells (Fig. 5D); even though cells were seeded on the same partly coated sample ensuring to be cultured under the same conditions.

**Figure 6:**
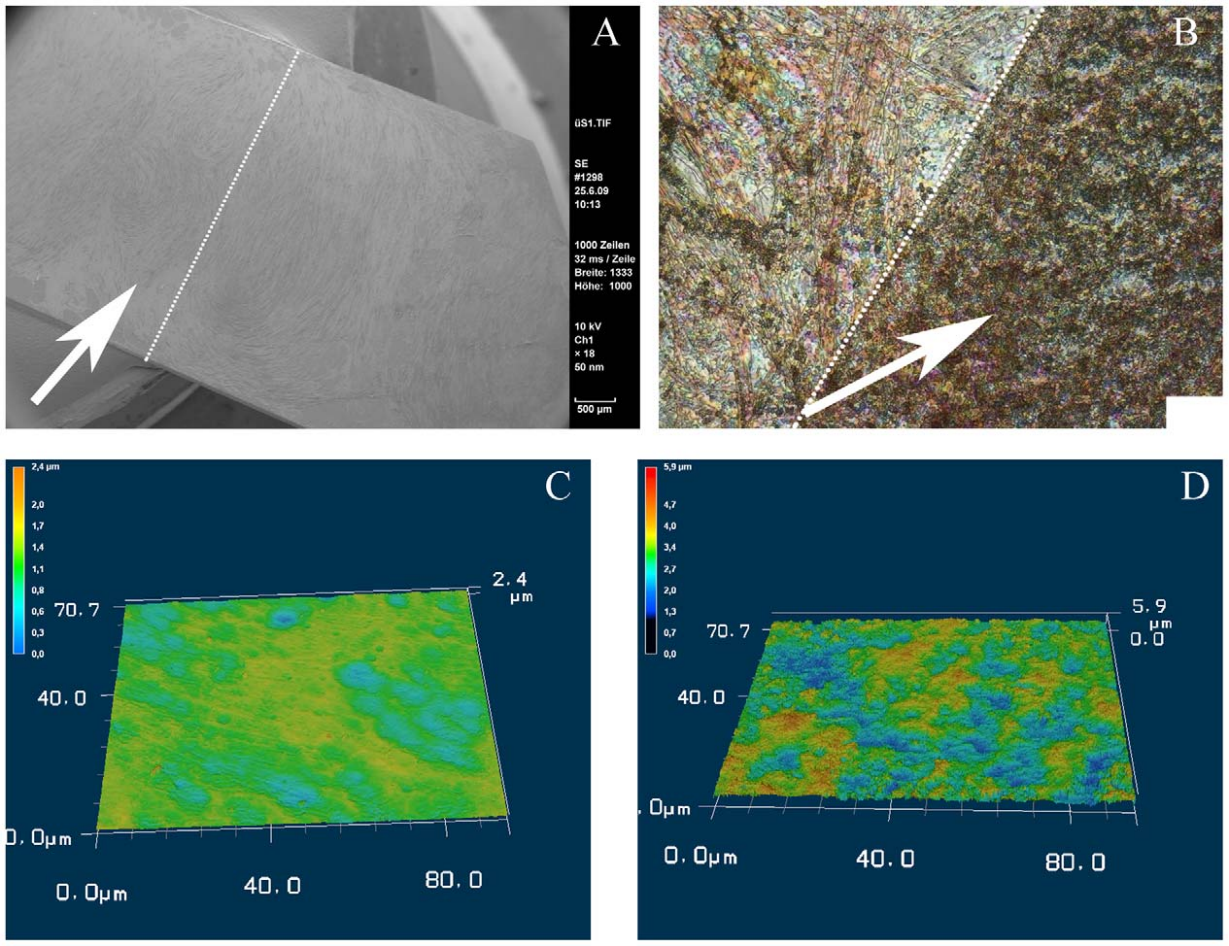
Matrix secretion of osteogenic ASCs on NiTi. SEM and violet laser scan of osteogenic ASCs revealed no visual differences in matrix secretion, whereas 3-d displays showed an enhanced morphology of cells and matrix on the coated parts of the sheets. A: SEM of half-coated NiTi-sheet (NiTi-nanoparticle coated part indicated by arrow, border indicated by dot line) with osteogenic ASCs; scale bar 500 µm. B: violet laser surface scan (VK-9700, Keyence) of half-coated NiTi-sheet (NiTi-nanoparticle coated part indicated by arrow, border indicated by dot line) with osteogenic ASCs; scale bar 10 µm. C: 3d-display of B, uncoated part. D: 3d-display of B, coated part.

SEM findings after 6 weeks of osteogenic stimulation showed large amounts of extracellular matrix secreted by the cells which grew densely on the NiTi-material. There was no apparent difference between coated and uncoated fractions of the Nitinol surfaces in this kind of analyses (Fig. 6A). A closer look at these samples by violet laser scanning microscopy of the surface showed a more flat shape of the extracellular matrix in contrast to a rough appearance of the matrix on the NiTi-nanoparticle coated half (Fig. 6B, 6C, 6D).

**Figure 7:**
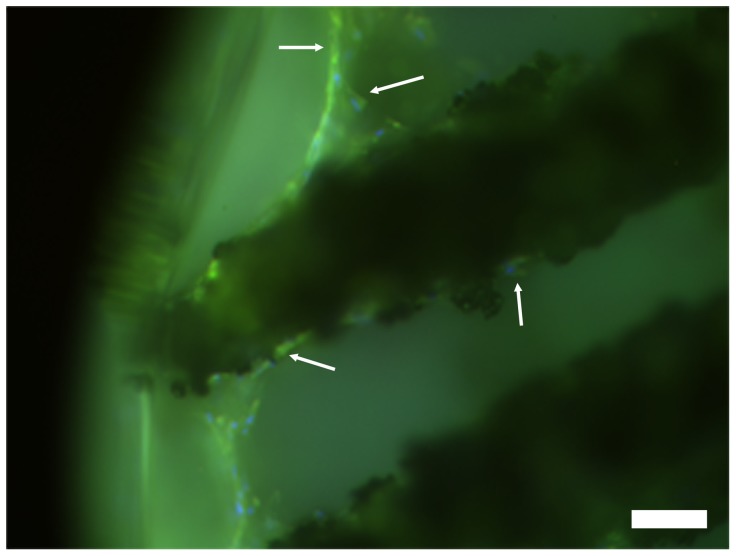
Osteogenic ASCs on NiTi-nanocoated NiTi secrete fibronectin as part of the extracellular matrix. blue: nuclei, green: fibronectin (indicated by arrows); scale bar 50 µm.

Fibronectin is known to be important for adhesion of osteoblasts on implant substrates in vitro [6]. In order to analyze adhesion of osteogenic ASCs, secreted fibronectin was detected by immunofluorescence. Therefore NiTi-nanoparticle coated NiTi-structures were seeded with ASCs and cultured under osteogenic conditions. After 6 weeks fibronectin was visualized via immunostainings. As shown in fig. 7 osteogenic ASCs on nanosurfaces secrete fibronectin.

**Figure 8:**
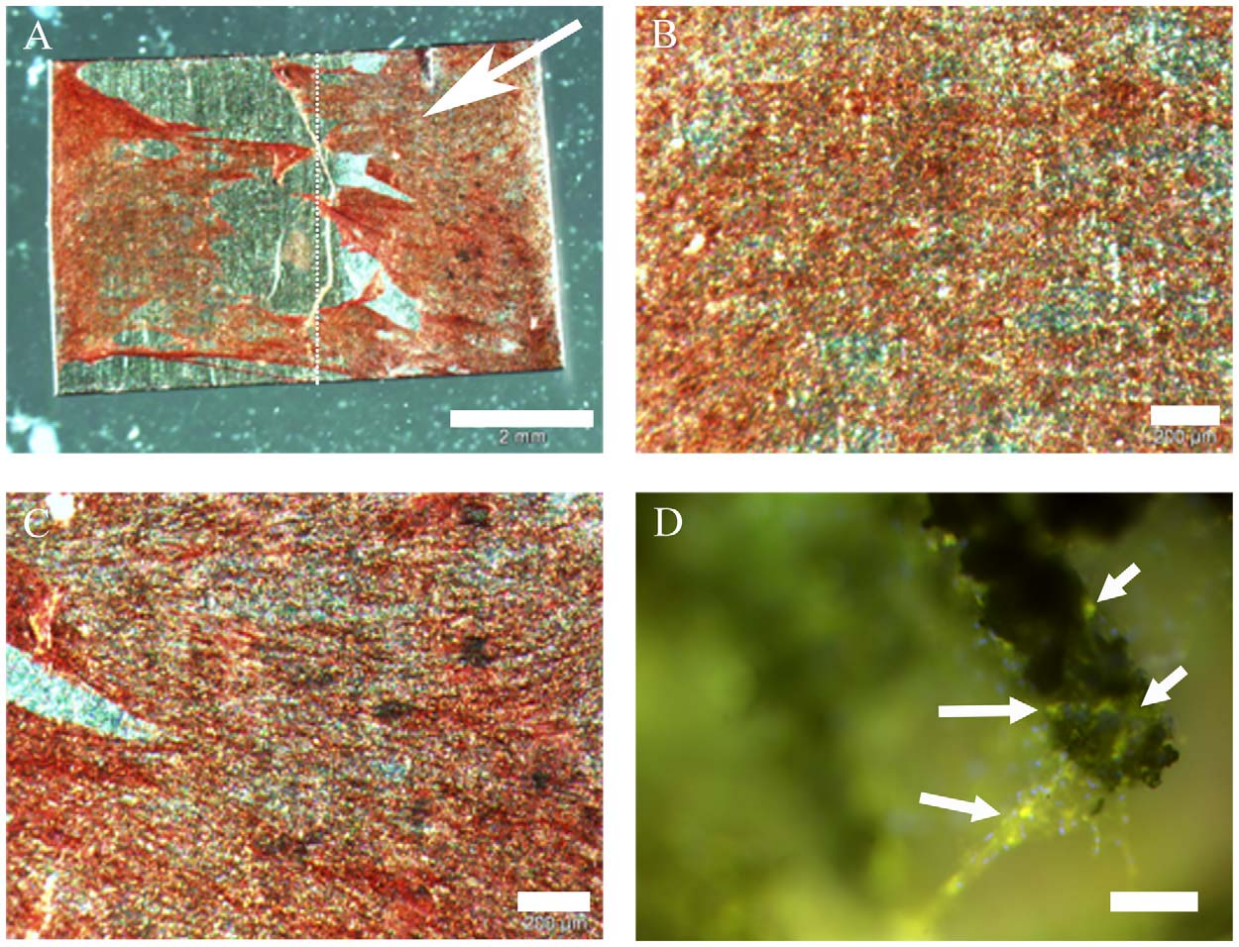
ASCs on NiTi, verification of osteogenic differentiation by staining of calcium depositions and osteocalcin. A: alizarin red staining, partly coated NiTi-sheet (coated part indicated by arrow, border indicated by dot line); scale bar 2 mm. B: detail of A, uncoated part; scale bar 200 µm. C: detail of A, coated part; scale bar 200 µm. D: NiTi-nanocoated structure with osteogenic ASCs; green: osteocalcin (indicated by arrows), blue: nuclei; scale bar 50 µm.

**Figure 9:**
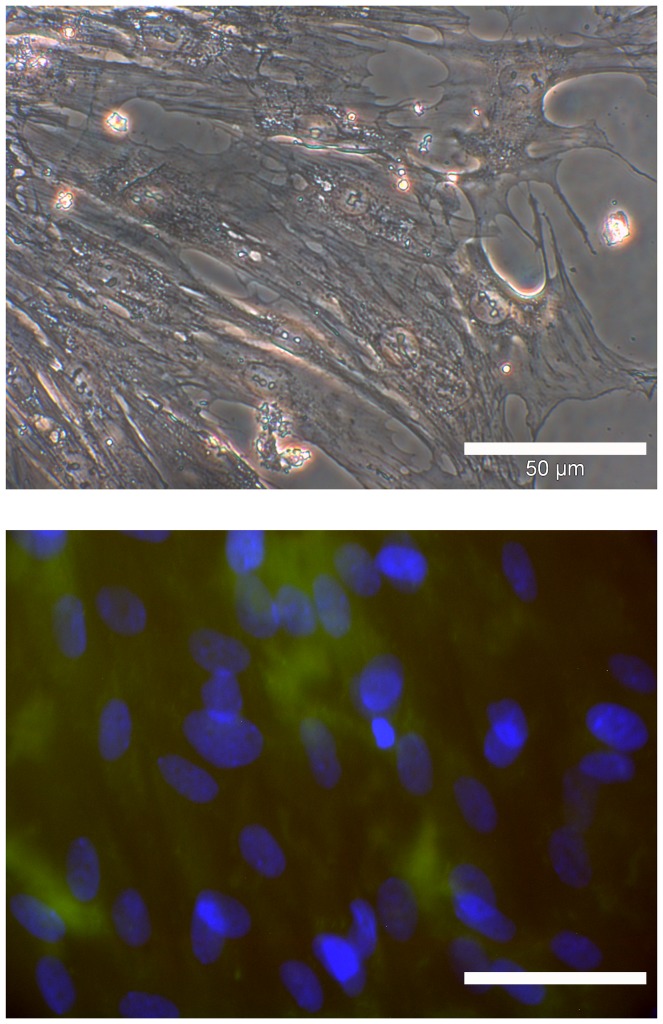
ASCs on cover glasses, 6 weeks cultivation without osteogenic medium. Alizarin red staining as well as immunofluorescence demonstrated that there did not occur osteogenesis or mineralization without osteogenic medium as the stainings were negative for the analyzed osteo markers. A: alizarin red staining; scale bar 50 µm. B: red: osteopontin, green: fibronectin, blue: nuclei; scale bar 50 µm.

**Figure 10:**
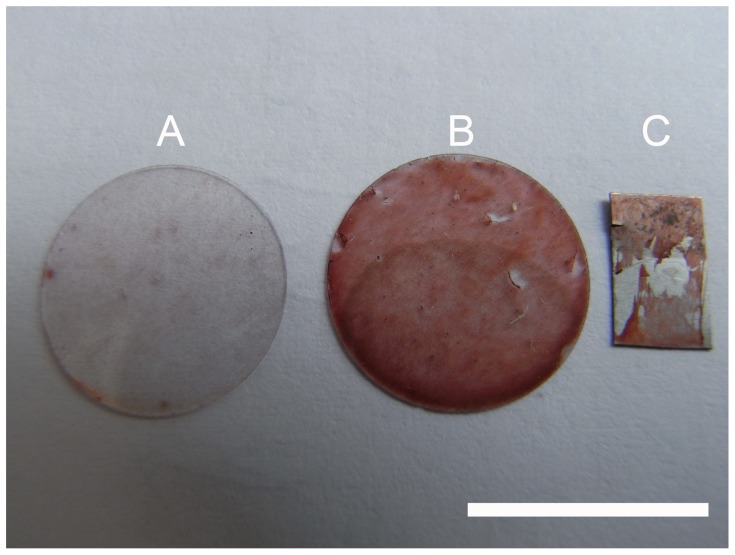
Macroscopic comparisons of alizarin red stainings. Comparison of stainings demonstrated that calcium deposition occurred only under long time cultivation of ASCs with osteogenic medium (B and C), while cells on A were kept under same conditions without osteogenic medium. Scale bar 1 cm. A: ASCs on cover glass, cultivated for 6 weeks with medium. B: ASCs on cover glass, cultivated for 6 weeks with osteogenic medium. C: ASCs on NiTi-sheet, cultivated for 6 weeks with osteogenic medium.

Specimens were stained with alizarin red for bone typical calcium depositions. Osteogenic samples were found to be positive (Fig. 8A, 8B, 8C). Further examination by immunofluorescence detected osteocalcin (Fig. 8D) and bone alkaline phosphatase (data not shown). No differences occurred between expression patterns on coated and uncoated surfaces. As undifferentiated ASCs do not express these markers, they served as negative control. Neither alizarin red stainings (Fig. 9A) nor immunofluorescences (Fig. 9B) showed calcium depositions or expression of osteogenic markers in cells cultivated with non-oestogenic medium for 6 weeks. Figure 10 compares alizarin red stainings macroscopically to demonstrate specificity. Calcium depositions were stained with the typical dark red color.

For all immunofluorescence stainings as an additional negative control nanoparticle-coated surfaces (glass and NiTi) without cells were treated after the same protocol to rule out false results due to unspecific adherence of antibodies to the coating. As shown by our results osteogenic differentiation could be induced chemically on uncoated and likewise on coated NiTi-surfaces.

## Discussion

### Toxicity

We could demonstrate that NiTi-nanoparticles coated on surfaces with a concentration up to 300 µg/cm^2^ as well as 2 µg/ml colloids did not cause toxic effects like lower metabolic activity or enhanced relative amounts of dead cells in cell cultures of ASCs and osteogenically differentiated ASCs. Relative amounts of living cell are comparable to control surfaces (glass, cell culture plastic, uncoated NiTi). These findings correlate with investigations of Hahn et al. regarding the in vitro compatibility of NiTi-nanoparticles with human coronary artery endothelial cells (hCAEC) and human coronary artery smooth muscle cells (hCASMC), which indicated that high concentrations of nanoparticles are needed to induce toxic reactions [11]. These toxic levels can not be achieved with the amount of particles which can be placed under the area of one single cell [12]. Also murine macrophages did not show reactions on NiTi-nanoparticles in viability assays. An increase of metabolic activity could have been an example for an immune reaction. As our data only give clues about in vitro effects of NiTi-nanoparticles, further experiments in vivo are necessary to ensure that particles do not induce negative reactions.

The influence of the uncoated control NiTi-sheets on the cells depends on the manufacturing processes. NiTi-sheets in this study were produced by rolling workpieces with 20 mm thickness. Surface finish was done by chemical etching, followed by rinsing steps with alcohol and water (manufacturer's information). Before application in cell culture sheets were autoclaved. According to the review of Shabalovskaya this treatments results in a better cytocompatibility, caused by a thicker oxide film (10–20 nm), a reduced Ni-surface concentration and a nearly negligible nickel release from the surface [24]. Based on this report, in our opinion the control NiTi-sheets used in our study allow an objective evaluation of benefits of the NiTi-nanoparticle coating. The question why more dead cells occurred on the uncoated sheets than on the coated ones after long time cultivation with osteogenic medium (Fig. 5) remained uncleared, but we think that this might depend on chemo-physical properties of the surfaces.

Recent studies raised the question of potential allergic reaction of NiTi. Jeswani et al. discussed the use of NiTi implants for intracranial stents. Based on clinical data, they concluded that the usage of NiTi stents for patients with known nickel allergies has to be considered carefully [25]. An in vitro study of Habijan showed that probably only a subpopulation of patients with nickel allergies reacts sensitive to NiTi [26]. Concerning to this data the allergic potential of NiTi-nanoparticles needs to be evaluated in detail by further studies.

### Cell adherence

The presented study demonstrated that NiTi-nanoparticles allow an increase of adhered cells when used for nanostructuring of Nitinol or glass surfaces. We tested coatings up to 300 µg/cm^2^ and noted increased numbers of adhered ASCs and an enhanced dimensional appearance. Muhonen et al. found differences in mouse osteoblast behavior on martensitic and austenitic NiTi, indicating that austenitic phase has better surface properties for cell adhesion. They discuss a lower hydrophobicity of austenit as a reason [27]. Accordingly, NiTi-nanoparticles decrease contact angles of the martensitic surface, which leads to a better wettability [12] and may cause the higher relative amounts of adhered ASCs.

The findings of enhanced ASC densities on nanocoatings also correlate with findings of Palin et al. who tested nanoscale structurings of Ti- and poly-lactic-co-glycolic acid moulds. They found enhanced CRL-11372 (human osteoblast cell line) densities on nanoscaled surfaces [28]. The review of Tran and Webster describes several studies which came to the conclusion that nanoscaled surfaces increase adhesion and function of osteoblasts as bone is a naturally nano-structured tissue [1]. Taken together the NiTi-nanoparticle coating not only enhances the wettability of martensitic NiTi, but also may help to mimic the nanofeatures of bone and therefore enhances the surface attractivity for cells.

Identified integrin α_5_- and β_1_-subunits as part of the adhesion of ASCs and osteogenic ASCs correlate with the knowledge about osteoblast adhesion on various substrates, where integrin α_5_ and β_1_ play an important role for the differentiation of those cells [6]. ASCs may behave similar to MSCs, which were already shown to differentiate osteogenic on untreated NiTi [14], [15]. Suitable to this type of integrin subunits we found high amounts of fibronectin (and thus the RGD-motif as ligand of integrin α_5_β_1_) – constituent part of the bone extracellular matrix – secreted by ASCs and osteogenic ASCs.

### Differentiation capacity

As ASCs showed enhanced relative adhesion rates and a dimensional growth it can be stated that the coating offers optimal conditions for the osteogenic differentiation of the cells. Honda et al. already demonstrated that three-dimensional growth promoted the differentiation of osteoblasts [29]. This group analyzed apatite-fiber scaffolds and demonstrated that the material itself also has an influence on the differentiation capacity of the cells. Of course the NiTi-nanoparticle coating has to be observed more in detail. According to Deligianni et al. who analyzed the influence of Ti-surface structure on human MSCs on osteogenic differentiation [30] we found good osteogenic differentiation of ASCs on Nitinol-nanostructures. Alizarin red stainings showed the typical calcium depositions. Also the osteogenic markers osteocalcin and bone alkaline phosphatase were detected. As important part of the extracellular matrix fibronectin was present in osteogenic samples. The expression of integrin α_5_- and β_1_-subunits was not visually changed by the nanocoatings, which also supports the conclusion that osteogenic differentiation capacity of ASCs is not changed by the adherence to nanoparticle coatings.

### Further aspects

The presented study was performed under static conditions, future trials should be repeated under cyclic loading according to the experimental set-up of Habijan [15] simulating the conditions after transplantation. This might give detailed information about possible Ni ion or nanoparticle release from the implant and allows studying cell reactions. Habijan tested commercially available dense NiTi cylindrical rods and found MSCs to survive on those even though there were slightly increased Ni concentrations in the cell culture media resulting from cyclic loading. As the surface of our NiTi-nanoparticle coated NiTi is larger, Ni release might be higher.

In vitro analyses already evaluated the possible corrosion of Nitinol specimens caused by osteoclasts which are an important part of healthy bone. No evidence for corrosion was found [26]. Accordingly, possible interactions between NiTi nanoparticles and osteoclasts should be analyzed before the coating is going to be evaluated in vivo.

According to Shabalovskaya's review the popular spray plasma technique to modify NiTi surfaces applies thermal stress to the material and thus is not suitable for implants with a complex geometry [24]. The deposition of nanoparticles might be an alternative method for surface modification without thermal stress.

The enhanced relative adherence rates of cells on the nanocoating in comparison to the uncoated Nitinol-surface in early times of cultivation (first 48 hrs) could be used in two potential ways: first is use of the NiTi-nanocoating for NiTi-implants to attract stem cells surrounding the place of defect and to accelerate the osseointegration of an implant according to Bahat et al. [31]. Second is the use of NiTi-nanocoating and autogenic ASCs to get a faster implant ingrowth as the ASCs tend to differentiate osteogenic when implanted in a bony surrounding [32] and may recruit vessels as ASCs have a high angiogenic potential [33], [34]. Furthermore, preseeding with autologous ASCs has another benefit: the co-transplantation of autologous material may decrease the probability of an inflammatory reaction. Several studies indicate mild immunosuppressive effects of ASCs in vitro and in vivo [35], [36], [37], [38].

### Conclusion

NiTi-nanoparticles up to a concentration to 300 µg/cm^2^ do not induce or cause any negative influence on cell appearance, abnormal proliferation or cell death of ASCs. The relative amounts of adhered cells are higher on NiTi-nanoparticle coatings compared to the bare material. As the osteogenic differentiation on NiTi-surfaces is not inhibited or negatively influenced, the combination of ASCs with NiTi-nanocoatings on NiTi-implant material offers new ways in implantology research regarding bioactivation by autologous stem cells and enhancement of surface attraction to cells, respectively.
